# Role of UPR Sensor Activation in Cell Death–Survival Decision of Colon Cancer Cells Stressed by DPE Treatment

**DOI:** 10.3390/biomedicines9091262

**Published:** 2021-09-18

**Authors:** Rossella Benedetti, Maria Saveria Gilardini Montani, Maria Anele Romeo, Andrea Arena, Roberta Santarelli, Gabriella D’Orazi, Mara Cirone

**Affiliations:** 1 Department of Experimental Medicine, La Sapienza University of Rome, Viale Regina Elena 324, 00185 Rome, Italy; rossella.benedetti@uniroma1.it (R.B.); mariasaveria.gilardinimontani@uniroma1.it (M.S.G.M.); mariaanele.romeo@uniroma1.it (M.A.R.); aarena026@gmail.com (A.A.); roberta.santarelli@uniroma1.it (R.S.); 2 Laboratory Affiliated to Istituto Pasteur Italia-Fondazione Cenci Bolognetti, 00185 Rome, Italy; 3 Department of Neurosciences, Imaging and Clinical Sciences, University “G. D’Annunzio”, 66013 Chieti, Italy; gdorazi@unich.it; 4 Unit of Cellular Networks, Department of Research and Advanced Technologies, IRCCS Regina Elena National Cancer Institute, 00144 Rome, Italy

**Keywords:** DPE, UPR, ATF6, colon cancer, p53

## Abstract

Polyphenols have been shown to possess several beneficial properties, including properties involved in the prevention or treatment of cancer. Among these polyphenols, a leading role is played by dihydroxyphenylethanol (DPE), the most powerful antioxidant compound contained in the olive oil. DPE has been previously reported to induce endoplasmic reticulum (ER) stress and to reduce cell survival in colon cancer, one of the most common and aggressive cancers in developed countries. In this study, we further investigated the activation of UPR by DPE and explored the roles of the three UPR sensors, inositol-requiring enzyme (IRE) 1 alpha, protein kinase RNA-like endoplasmic reticulum kinase (PERK), and activating transcription factor (ATF6), in the cell death–survival decision of wt and mutp53 colon cancer cells and the underlying mechanisms involved. We also unveiled a new interplay between ATF6 and wt, as well as mutp53, which may have important implications in cancer therapy.

## 1. Introduction

The unfolded protein response (UPR) is a defense mechanism that helps cells to face endoplasmic reticulum (ER) stress. GRP78, also called BiP or HSPA5, an ER chaperone belonging to heat shock protein (HSP) 70 family, is the master regulator of the UPR. In normal conditions, its binding to the three UPR sensors, namely inositol-requiring enzyme (IRE) 1 alpha, protein kinase RNA-like endoplasmic reticulum kinase (PERK), and activating transcription factor (ATF6), maintains them in an inactive state. When unfolded or misfolded proteins accumulate in the ER causing ER stress, they attract BiP and detach it from UPR sensors, resulting in UPR activation [1]. Cancer cells are basally stressed, given their elevated protein synthesis, rapid growth, nutrient shortages, state of hypoxia, and the presence of oncogenes. The intensity and duration of ER stress dictate the outcome of UPR in terms of cell survival or death, as it may promote pro-survival processes such as protein degradation and protein translation inhibition, it may upregulate chaperones such as BiP, or in cases of mild stress it may lead to an increased expression of CHOP and apoptosis, such as when the protective capacities of UPR are overwhelmed. The basal stress that characterizes cancer cells offers the possibility to exacerbate ER stress and selectively kill these cells while sparing normal cells; however, cancer cells may still attempt to resist treatments exacerbating ER stress by further upregulating BiP, which sustains cell proliferation and counteracts apoptosis [2]. Indeed, targeting BiP via specific inhibitors may represent an effective anticancer strategy [3] and an opportunity to overcome the resistance of cancer cells to treatments inducing ER stress [4], which may be the cause of incomplete tumor elimination and tumor relapse after therapies. Lastly, the manipulation of UPR may influence the immune response against dying cancer cells. For example, PERK/eIF2alpha activation has been reported to induce the translocation of calreticulin on cell surfaces via an “eat me” signal activating dendritic cells [5,6], while the upregulation of CHOP may increase the release of PGE2, which in turn triggers ER stress in DCs, rendering them dysfunctional [7]. Among the treatments able to activate ER stress or UPR in cancer cells are natural compounds such as polyphenols [8]. The main advantage of the use of these compounds is the lack of side effects towards normal cells, including those belonging to the immune system, whose function is required for complete tumor eradication [9]. One such polyphenol that has been reported to induce ER stress by perturbing Ca^2+^ homeostasis is dihydroxyphenylethanol (DPE), also called hydroxytyrosol [10], a component of olive oil, which is a key component of the Mediterranean diet. Besides DPE, olive oil contains several other phenolic compounds displaying antioxidant and anti-inflammatory properties, such as oleuropein and tyrosol [11]. ER stress and UPR activation caused by DPE has been shown to impair cell survival in colon cancer cells. This cancer represents a leading cause of death in industrialized countries, as its onset is caused by the unhealthy lifestyle and diet. Moreover, there are different forms of inherited colorectal cancers, such as non-polyposis colorectal cancer (HNPCC) and familial adenomatous polyposis (FAP) [12]. Despite the efforts made so far, the response of colon cancer to chemotherapies in advanced states is quite poor [13]. Although the above reported study showed that DPE impaired cell survival in colon cancer cells carrying mutant (mut) and wild-type (wt) p53, most of the experiments were performed only in a mutp53 cell line, in which DPE activated the PERK/eIF2alpha/CHOP axis and the phosphatase PP2A, de-phosphorylating ERK1/2 and AKT [10]. Here, we further investigate the UPR activation caused by DPE and evaluate its impact on the activation of IRE1 alpha, PERK, and ATF6, as well as the role of the signaling initiated by these sensors on the expression of BiP and CHOP, and ultimately in cell death or survival. As all experiments are conducted in parallel in colon cancer cells harboring wtp53 and mutp53, the impact of UPR sensor activation on wt and mutp53 expression is also investigated.

## 2. Materials and Methods

### 2.1. Cell Cultures and Treatments

RKO wtp53, SW480 mutp53, HCT116 wtp53, and HCT116 p53-/- were maintained in DMEM 1640 (Thermo Fisher Scientific) supplemented with 10% fetal bovine serum (FBS) (Corning), L-glutamine, streptomycin (100 μg/mL) (Corning), and penicillin (100 U/mL) (Corning) (complete medium) in 5% CO_2_ at 37 °C. Human colonic epithelial cells (HCoEpC) were cultured in epithelial cell growth medium (iXCells Biotechnologies USA, MD-0041). Cells were always detached using a trypsin–EDTA solution (Biological Industries, Cromwell, CT, USA). 

Cells were plated in 12-well plates at a density of 10^5^ cells/well in 1 mL, then the day after were treated with 3,4-dihydroxyphenyl ethanol (DPE) (25 μM, 50 μM, 100 μM, 200 μM) for 24 or 48 h. To evaluate the expression levels of proteins in living cells and dead cells, after 48 h of treatment with DPE (50 or 100 μM), cells in suspension, representing the fraction of dead cells, were separated from those cells that were alive and still adherent to the plate. Living cells were washed in 1× PBS, detached using trypsin–EDTA solution, and pelleted separately. Some experimental cells were plated in 12-well plates, as reported above, then the day after were pre-treated with HA15 (BiP/GRP78 inhibitor) (Sigma-Aldrich, MO, USA, SML2118), 4μ8C (IRE1 RNAse inhibitor) (Sigma-Aldrich, Saint Louis, MO, USA, SML0949), ceapin-A7 (ceapin) (ATF6a signaling blocker) (Sigma-Aldrich, MO, USA, SML2330), or GSK2606414 (GSK) (PERK inhibitor) (Selleckem, Houston, TX, USA, S7307). Chemicals were added to cell cultures at the final concentrations of 10 μM (HA15), 10 μM (4μ8C), 12 μM (Ceapin), and 0.5 μM (GSK2606414) for 1 h, then treated with DPE (50 μM) and cultured for the next 48 h. Untreated cells were used as controls. Each experiment was performed in triplicate and repeated at least three times.

### 2.2. Cell Viability

Cell viability was evaluated via a trypan blue (Sigma-Aldrich, MO, USA) exclusion assay after 24 or 48 h of culture. Cells were counted via light microscopy using a Neubauer hemocytometer. The experiments were performed in triplicate and repeated at least three times.

### 2.3. Cell Proliferation

Cell proliferation was evaluated by MTT assay (Sigma Aldrich). Here, 5 × 10^3^ cells/well were plated in 96-well plates in 100 μL of complete medium. The next day, cells were treated with different doses of DPE (25 μM, 50 μM, 100 μM, 200 μM, 400 μM) for 24 h. Untreated cells were used as controls. The MTT assay was performed following the manufacturer’s instruction. The plates were analyzed using an Absorbance 96 reader (Byonoy GmbH). The experiments were performed in triplicate and repeated three times. 

### 2.4. Cell Cycle Analysis 

For cell cycle analysis, the DNA contents of untreated or treated RKO and SW480 cell lines were measured using propidium iodide (PI, Sigma Aldrich) staining and FACS analysis. After 24 or 48 h of treatment with DPE 50 or 100 μM or control, cells were washed with cold 1× PBS and fixed in 70% ethanol on ice for at least 1 h. After centrifugation, the cell pellet was washed with cold 1× PBS, stained with 50 μg/mL PI and RNase for 15 min at 37 °C, then analyzed using FACSCalibur (BD Biosciences). Data are representative of at least three independent experiments. 

### 2.5. Western Blot Analysis

Following treatments, cells were washed in 1× PBS, lysed in RIPA buffer (150 mM NaCl, 1% NP-40, 50 mM Tris-HCl (pH 8), 0.5% deoxycholic acid, 0.1% SDS, protease, and phosphatase inhibitors), then centrifuged at 14,000 rpm for 20 min at 4 °C. The protein concentration was measured using the Bio-Rad Protein Assay (BIO-RAD laboratories GmbH) and 15 μg of protein was subjected to electrophoresis on 4–12% NuPage Bis-Tris gels (Life Technologies) according to the manufacturer’s instructions. The gels were transferred to nitrocellulose membranes (Biorad, Hercules) for 1.5 h in Tris–glycine buffer and the membranes were blocked in 1× PBS-0.1% Tween20 solution containing 3% of BSA (Serva), probed with specific antibodies, and developed using ECL Blotting Substrate (Advansta). 

### 2.6. Antibodies 

To evaluate protein expression on Western blot membranes, the following antibody were used: rabbit polyclonal anti-ATF6 (1:600) (Proteintech, 24169-1-AP), rabbit polyclonal anti-XBP1 (1:1000) (Novus Biologicals, NB100-80861), rabbit polyclonal anti-phospho-eIF2alpha (Ser51) (1:1000) (Cell Signaling, 9721), rabbit polyclonal anti-eIF2alpha (1:4000) (Cell Signaling, 9722), rabbit polyclonal anti-BiP/GRIP78 (1:5000) (Proteintech, 11587-1-AP), rabbit polyclonal anti-CHOP (GADD153) (1:1000) (Proteintech, 15204-1-AP), mouse monoclonal anti-DUSP5 (1:200) (Santa Cruz Biotechnology Inc., Dallas, TX, USA sc-393801), mouse monoclonal anti-p-ERK (Santa Cruz Biotechnology Inc., sc-7383), rabbit polyclonal anti-ERK1 (Santa Cruz Biotechnology Inc., sc-93), rabbit polyclonal anti-ERK2 (Santa Cruz Biotechnology Inc., sc-154), rabbit monoclonal anti-Mcl1 (1:1000) (Cell Signaling, 39224), mouse monoclonal anti-p53 (1:100) (clone DO-1, Santa Cruz Biotechnology Inc., sc-126). Mouse monoclonal anti-β-actin (1:10,000) (Sigma Aldrich) was used as the loading control. The goat antimouse IgG-HRP (1:30,000) (Bethyl Laboratories, A90-116P) and goat antirabbit IgG-HRP (1:30,000) (Bethyl Laboratories, A120-101P) were used as secondary antibodies. All primary and secondary antibodies were diluted in PBS–0.1% Tween20 solution containing 3% of BSA (SERVA).

### 2.7. Statistical Analysis

The results are represented by the means ± standard deviation (S.D.) of at least three independent experiments, while a two-tailed Student’s *t*-test was used to demonstrate statistical significance. A difference was considered as statistically significant when the *p*-value was at least <0.05.

## 3. Results

### 3.1. DPE Impairs Cell Survival and Proliferation in a Dose-Dependent Fashion in WT and Mutp53-Carrying Colon Cancer Cells While Sparing Normal Colon Epithelial Cells

**Figure 1:**
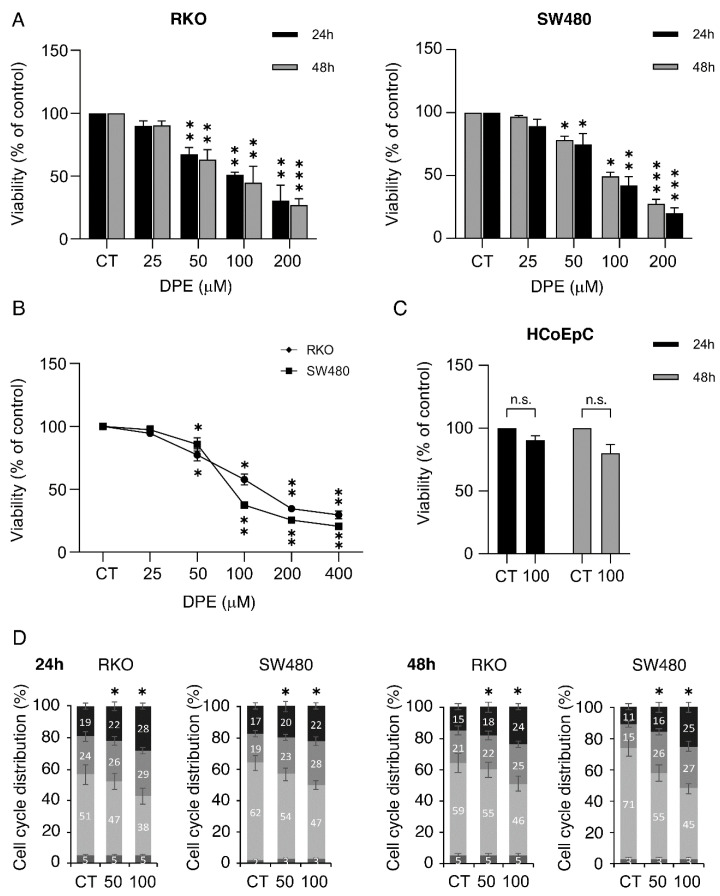
Dose–time–response inhibition of proliferation and S-G2 cell cycle arrest induction by DPE in colon cancer cells: (**A**) cell viability of RKO and SW480 cell lines treated with different doses of DPE (25, 50, 100 and 200 μM) or left untreated as control (CT), as measured by a trypan blue exclusion assay after 24 or 48 h of treatment; (**B**) cell proliferation of RKO and SW480 cell lines treated with different doses of DPE (25, 50, 100, 200 μM and 400 μM) or control, as measured by MTT assay after 24 h; (**C**) cell viability of human colonic epithelial cell (HCoEpC) lines treated with DPE (100 μM) or left untreated as control, as measured by a trypan blue exclusion assay after 24 or 48 h of culture; (**D**) cell cycle analysis of RKO and SW480 treated with DPE (50 or 100 μM) or untreated for 24 or 48 h, as evaluated by FACS analysis after staining with PI. One representative out of three experiments is shown. The bars represent the means of the percentages of cells in each phase of the cell cycle (subG1, G1, S, and G2) plus S.D. of three experiments. Note: *** *p* < 0.001, ** *p* < 0.01, * *p* < 0.05. Not significant (n.s.).

RKO wtp53 and SW480 mutp53 colon cancer cells were treated with different doses of DPE (50 and 100 μM) for 24 and 48 h, then cell survival and proliferation were evaluated via trypan blue and MTT assays, respectively. As shown in Figure 1A,B, DPE effectively reduced survival in a dose- and time-dependent fashion in both wtp53 RKO and mutp53 SW480 cells, although the former cells were more susceptible to the lowest doses of DPE. We then found that DPE reduced RKO and SW480 cell proliferation in a dose-dependent fashion by performing an MTT assay (Figure 1B). Interestingly, DPE slightly affected cell proliferation of normal epithelial colon cells (HCoEpC) (Figure 1C), suggesting that it can be used safely. Next, to further explore the impact of DPE on the reduction of cell proliferation, a cell cycle analysis was performed. As shown in Figure 1D, DPE induced S-G2 cell cycle arrest after 24 and 48 h of treatment.

### 3.2. DPE Activated All UPR Branches and Induced Dose-Dependent Upregulation of CHOP

**Figure 2:**
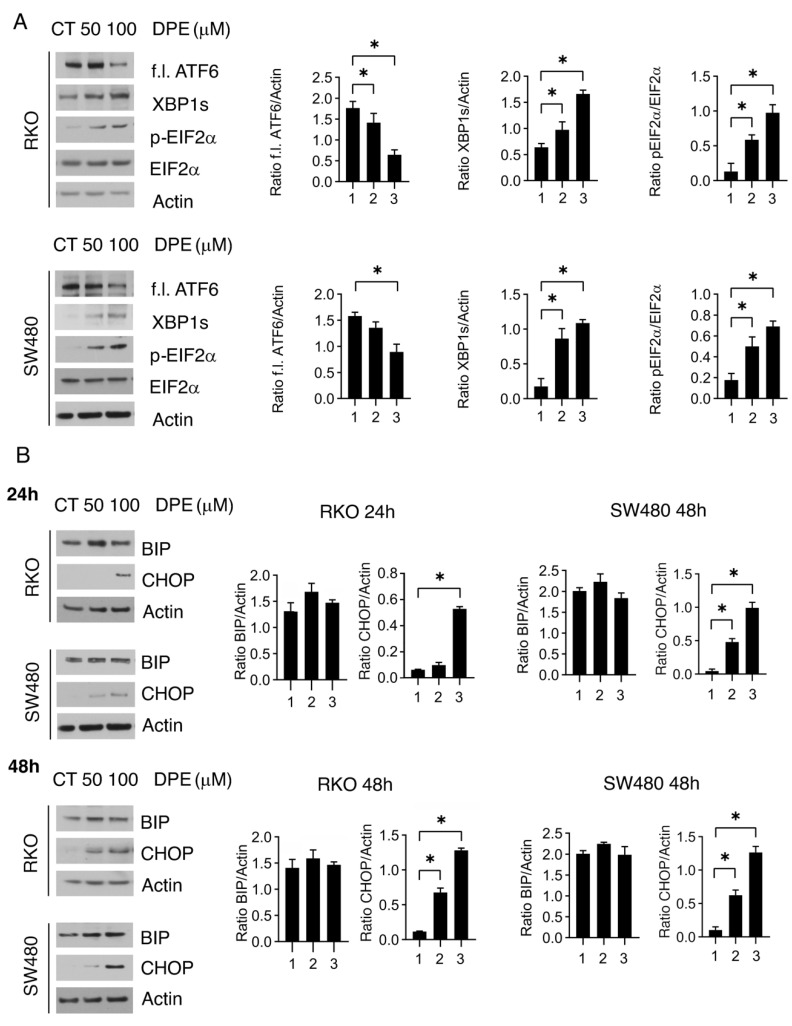
DPE induces ER stress and activates the pro-apoptotic transcription factor CHOP. (**A**) RKO and SW480 were treated with DPE (50 or 100 μM) or left untreated as control. After 24 h of treatment, the expression levels of ATF6, XBP1s, p-EIF2a, and EIF2a were evaluated via Western blot analysis. β-Actin was used as the loading control. The histograms represent the means plus S.D. from the densitometric analysis of the ratio between the protein and the appropriate control. Note: * *p* < 0.05. (**B**) Expression levels of BiP and CHOP were evaluated via Western blot analysis after 24 or 48 h of treatment with DPE (50 or 100 μM) or control. β-Actin was used as the loading control. The histograms represent the means plus S.D. from the densitometric analysis of the ratio between the protein and β-actin. Note: * *p* < 0.05.

We next assessed the impact of DPE on the activation of all three main UPR sensors, IRE1alpha, PERK, and ATF6 in both wtp53 RKO and mutp53 SW480 cell lines. As shown in Figure 2A, the ATF6 full-length form was reduced, as occurs when it is activated. Indeed, in the course of ER stress, ATF6 translocates from ER to the Golgi apparatus where it undergoes proteolysis and becomes an activated transcription factor [14]. Moreover, the spliced form of XBP1, resulting from IRE1alpha endoribonuclease activity, also increased in DPE-treated cells and finally PERK was activated, as the phosphorylation of its target eIF2alpha increased. These results suggest that all UPR sensors were activated by DPE, both in RKO and SW480 cells. Depending on the intensity and duration of stress, UPR may induce a reduction of cell survival, i.e., by upregulating CHOP, or help cells to adapt to stress by upregulating BiP [15]. Here, we found that the expression level of CHOP increased in a dose- and time-dependent fashion in both cancer cell lines (Figure 2B), in correlation with the impairment of cell survival observed at higher doses. Differing from CHOP, the expression of BiP was slightly affected by DPE treatment in both cell lines (Figure 2B). 

### 3.3. DPE Induces ERK1/2 De-Phosphorylation and DUSP5 Upregulation

**Figure 3:**
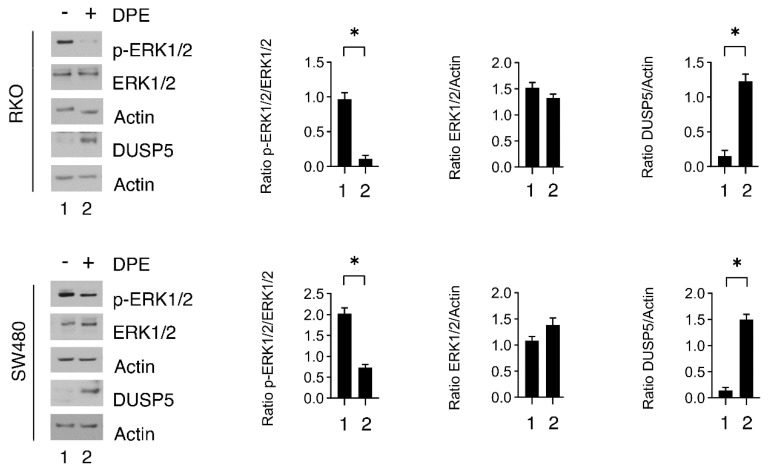
DPE upregulated DUSP5 and induces p-ERK dephosphorylation. RKO and SW480 were treated with DPE (50 μM) or left untreated as controls. After 24 h, the expression levels of p-ERK1/2, ERK1/2, and DUSP5 were evaluated via Western blot analysis. β-Actin was used as the loading control. The histograms represent the means plus S.D. from the densitometric analysis of the ratio between the protein and the appropriate control. Note: * *p* < 0.05.

CHOP has been reported to contribute to the activation of PP2A, which has been shown to mediate de-phosphorylation of AKT and ERK1/2 following DPE treatment [10]. Accordingly, here we found that after 48 h of treatment, the time at which CHOP was upregulated, ERK1/2 phosphorylation was reduced, particularly in RKO cells (Figure 3). DPE increased the expression level of DUSP5 (Figure 3), a phosphatase that has been shown to be upregulated by CHOP [16] and to directly mediate the de-phosphorylation of ERK1/2 [17]. This suggests that under intense ER stress, ERK1/2 may be targeted by several phosphatases to reduce its activation and induce cell death. This is not surprising, considering that ERK1/2 is a kinase that has been shown to prevent apoptosis during ER stress [18]. 

### 3.4. BiP Is Upregulated in the Fraction of Colon Cancer Cells Resistant to DPE and Targeting BiP May Improve the Outcome of DPE-Treatment

**Figure 4:**
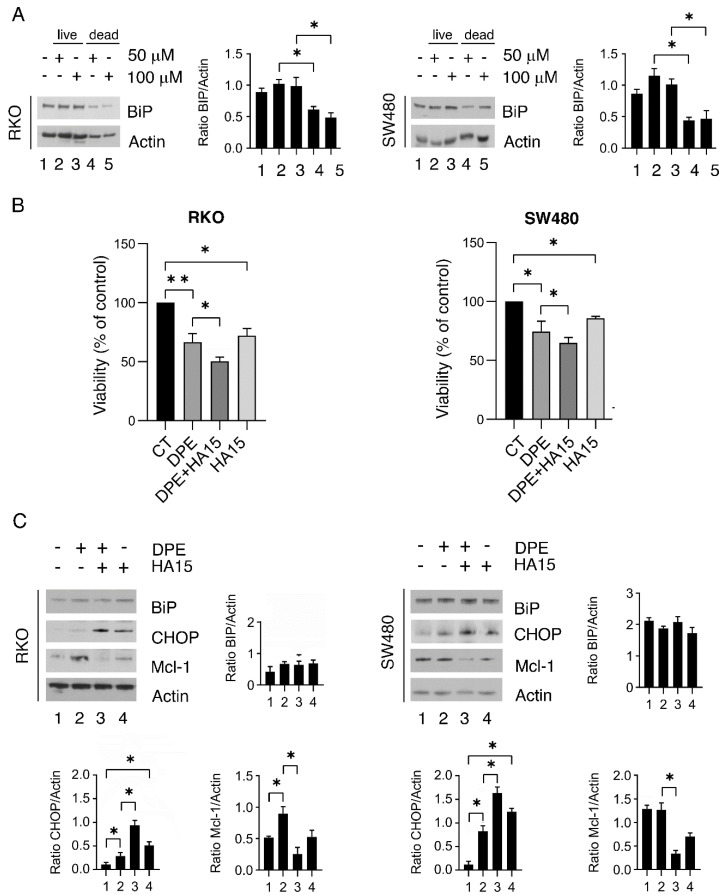
BiP is upregulated in the fraction of cells resistant to the DPE treatment. (**A**) RKO and SW480 were treated with DPE (50 or 100 μM) or left untreated as controls. After 48 h, the expression of BiP in living cells or dead cells was evaluated via Western blot analysis. β-Actin was used as the loading control. The histograms represent the means plus S.D. from the densitometric analysis of the ratio between BiP and β-actin. (**B**) The cell viability levels of RKO and SW480 cell lines pre-treated with HA15 (10 μM) and then treated with DPE (50 μM) or left untreated as control (CT) were measured via trypan blue exclusion assay after 48 h of culture. (**C**) RKO and SW480 were pre-treated with HA15 (10 μM) and then treated with DPE (50 μM) or left untreated as control. After 48 h, the expression levels of BiP, CHOP, and Mcl-1 were evaluated via Western blot analysis. β-Actin was used as the loading control. The histograms represent the means plus S.D. from the densitometric analysis of the ratio between the protein and β-actin. Note: ** *p* < 0.01, * *p* < 0.05.

As mentioned above, cancer cells attempt to resist treatments that exacerbate ER stress by further upregulating BiP. This indeed helps protein re-folding into the ER and sustains cell survival [2]; therefore, we isolated the fraction of cells that were resistant to the treatment and that still remained attached to the bottoms of the wells to assess the expression of BiP. As shown in Figure 4A, BiP was more expressed in the populations of live cells compare to those of mixed or dead cells. We next investigated whether the targeting of BiP by a specific inhibitor, HA15, could potentiate the cytotoxic effect mediated by DPE. We found that HA15 that was cytotoxic against colon cancer cells and further increased the cytotoxicity of DPE (Figure 4B). This effect correlated with a higher expression level of CHOP and the downregulation of the antiapoptotic molecule Mcl-1 (Figure 4C), a Bcl-2 family protein, whose expression can be modulated by CHOP [19]. Importantly, DPE alone, when used at low doses, slightly increased Mcl-1 expression (Figure 4C).

### 3.5. UPR Sensor Inhibition Differently Affects Cell Survival in DPE-Treated Colon Cancer Cells 

The UPR sensors trigger independent and also inter-connected signaling pathways from whose interplay cell survival/death decision depends. PERK/eIF2alpha, that is the most important UPR axis in promoting cell death, through the upregulation of CHOP, can also activate pro-survival pathways such as NRF2 [20] and STAT3 [21] and may strongly contribute to triggering the autophagic process [22]. ATF6, which upregulates chaperones such as BiP [23], may also play a role in increasing the expression of CHOP [24], and finally IRE1alpha, as its endo-ribonuclease activity induces the splicing of XBP-1 (XBP1s) and adaption to stress, which can also promote apoptosis by promoting the phosphorylation of JNK [25]. 

**Figure 5:**
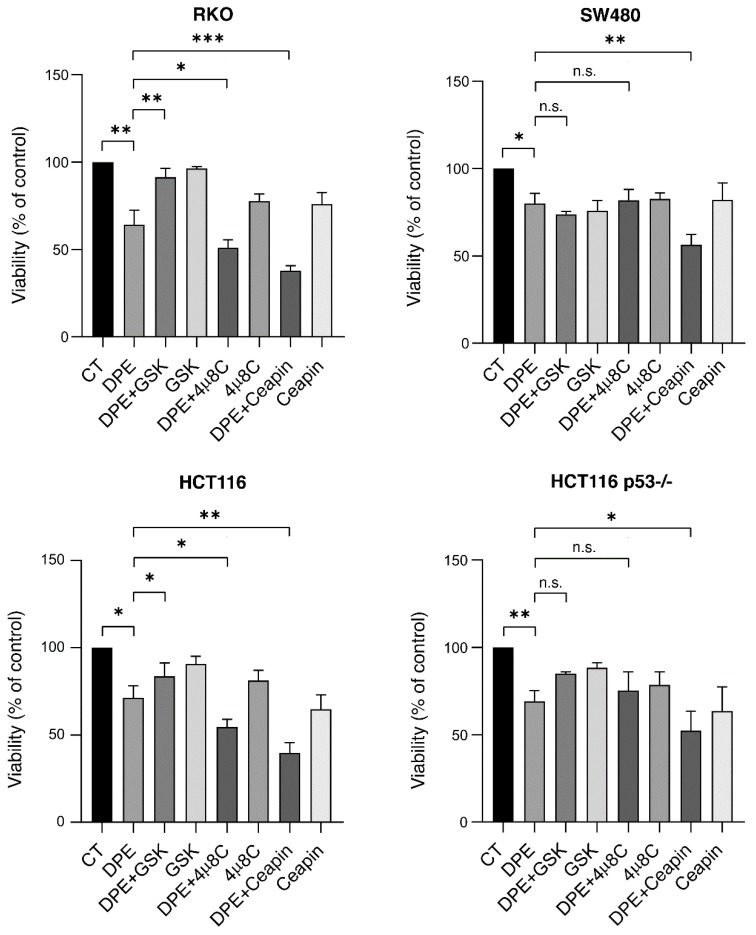
UPR sensor inhibitors differently affect cell survival of DPE-treated cells. Cell viability levels of RKO, SW480, HCT116, and HCT116 p53-/- cell lines pre-treated with 4μ8C (10 μM), GSK (0.5 μM), or ceapin (12 μM) and then treated with DPE (50 μM) or left untreated as control (CT), as measured via trypan blue exclusion assay after 48 h of culture. Note: *** *p* < 0.001 ** *p* < 0.01, * *p* < 0.05. Not significant (n.s.).

Given this intricate interplay, we inhibited the UPR sensors using specific inhibitors to evaluate their role in cell death and survival in RKO and SW480 cells stressed by DPE treatment. We extended this study and evaluated the role of UPR sensor activation in other colon cancer cells carrying wt or dysfunctional p53, namely wtp53 HCT116 and p53-/-. As shown in Figure 5, PERK inhibition by GSK2606414 (GSK) partially counteracted the reduction of cell survival induced by DPE, while 4u8c, an inhibitor of IRE1 alpha endoribonuclease activity, potentiated DPE-mediated cytotoxic effects in wtp53 RKO and HCT116 cells. Both GSK and 4u8c slightly affected the cell survival of SW480 and HCT116 p53-/- cells undergoing DPE-treatment (Figure 5). We finally investigated the role of ATF6 by using its specific inhibitor ceapin [26] and found that it potentiated the cytotoxic effects of DPE more efficiently than 4u8c in RKO and HCT116 wtp53 (Figure 5). It was also effective against SW480 cells and HCT116 p53-/- (Figure 5). 

### 3.6. GSK2606414 Downregulates CHOP and Upregulates Mcl-1 While 4u8c Induces Opposite Effects 

**Figure 6:**
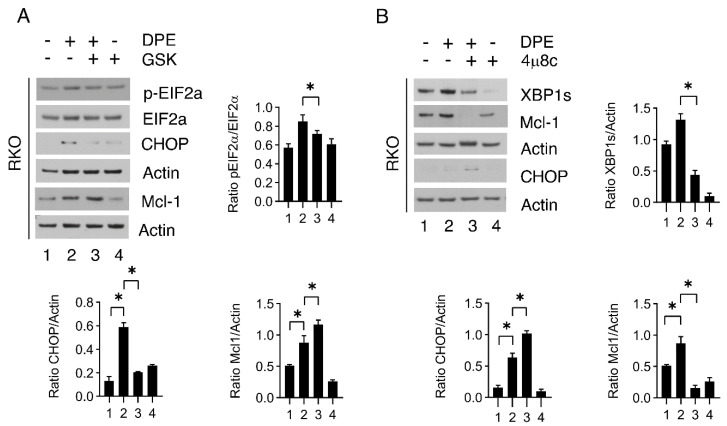
Inhibition of PERK or IRE1alpha influences the expression of CHOP and Mcl-1. (**A**) RKO was pre-treated with GSK (0.5 μM) and then treated with DPE (50 μM) or left untreated as control. After 48 h, the expression levels of p-EIF2α, EIF2α, CHOP, and Mcl-1 were evaluated by Western blot analysis. β-Actin was used as the loading control. The histograms represent the means plus S.D. from the densitometric analysis of the ratio between the protein and the appropriate control. (**B**) RKO was pre-treated with 4μ8C (10 μM) and then treated with DPE (50 μM) or left untreated as control. After 48 h, the expression levels of XBP1s, CHOP, and Mcl-1 were evaluated via Western blot analysis. β-Actin was used as the loading control. The histograms represent the means plus S.D. from the densitometric analysis of the ratio between the protein and β-actin. Note: * *p* < 0.05.

We further explored the effects of GSK and 4u8c in RKO, cells in which these inhibitors reduced or potentiated the cytotoxic effects of DPE, respectively. We first assessed the capacity of GSK to effectively reduce eIF2alpha phosphorylation and then evaluated its impacts on CHOP and Mcl-1 expression. As shown in Figure 6A, the phosphorylation of eIF2alpha and CHOP expression, which was slightly increased by DPE, was reduced by GSK. On the other hand, Mcl-1 was more expressed in DPE/GSK-treated cells. These mechanisms may underlie the reduction in DPE cytotoxicity via combination with GSK. Next, we found that 4u8c efficiently inhibited XBP1s accumulation, which conversely to GSK further increased CHOP and dramatically reduced Mcl-1 expression (Figure 6B).

### 3.7. Atf6 Inhibition by Ceapin Reduces Bip Expression Levels Both in Rko and sw480 Cells and Stabilizes wtp53 in the Former While Downregulating mutp53 in the Latter

**Figure 7:**
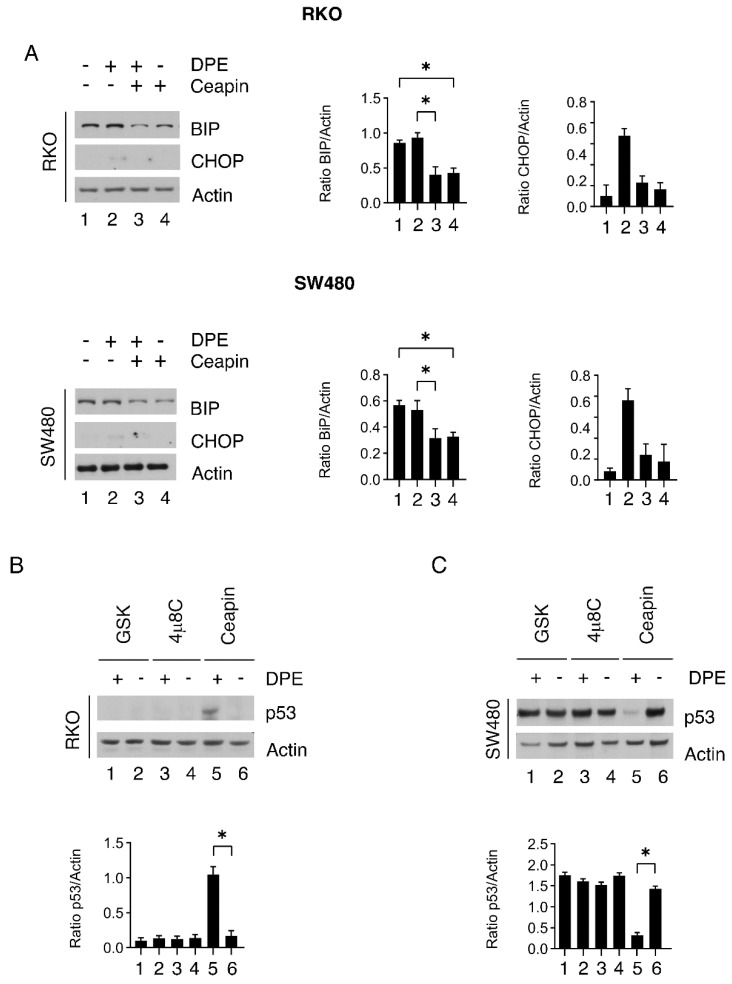
Inhibition of ATF6α reduces BiP expression and leads to wtp53 activation or mut p53 reduction in wild-type and mutp53 cells, respectively. (**A**) RKO and SW480 were pre-treated with ceapin (12 μM) and then treated with DPE (50 μM) or left untreated as control. After 48 h, the expression of BiP and CHOP was evaluated by Western blot analysis. β-Actin was used as the loading control. The histograms represent the means plus S.D. of the densitometric analysis of the ratio between the protein and β-actin. RKO (**B**) and SW480 (**C**) were pre-treated with 4μ8C (10 μM), GSK (0.5 μM), or ceapin (12 μM) and then treated or not with DPE (50 μM). After 48 h, the expression of p53 was evaluated via Western blot analysis. β-Actin was used as the loading control. The histograms represent the means plus S.D. of the densitometric analysis of the ratio between the protein and β-actin. Note: * *p* < 0.05.

Searching for the molecular mechanisms leading to the potentiating effect induced by ceapin in combination with DPE, we found that this ATF6 inhibitor downregulated BiP (Figure 7A), according to the role of ATF6 in upregulating its expression [23]. Differently from BiP, CHOP was not upregulated by ceapin (Figure 7A), possibly because ATF6 contributes to CHOP upregulation [24]. To further investigate the mechanisms leading to the cytotoxic effect of ceapin in combination with DPE, we found that wtp53 was stabilized in RKO cells (Figure 7B), while mutp53 was downregulated in SW480 cells (Figure 7C). Both wtp53 activation and mutp53 reduction may lead to reductions in cell proliferation in cancer-carrying wt and mutp53, respectively [27,28]. These mechanisms could, thus, play a role in the cytotoxic effect mediated by the combination of ceapin and DPE in wtp53- and mutp53-carrying cells.

## 4. Discussion

In this study, we explored UPR sensor activation and its role in the cytotoxic effect of DPE against wt and mutp53 colon cancer cells. The choice of this cancer type was due to the fact that DPE is a component of olive oil, meaning it is ingested with food and directly interacts with colon epithelial cells. For the same reason, we also explored and proved the safety of DPE against normal epithelial colon cells. We showed that DPE induced cell cycle arrest in both wt and mutp53 colon cancer cells, although the latter cells were slightly less responsive to the treatment. The reduction of cell proliferation induced by DPE correlated with the activation of all UPR sensors, from the interplay of which CHOP was upregulated and ERK1/2 was de-phosphorylated. BiP was upregulated mainly in the fraction of cells resistant to DPE treatment, and accordingly its targeting by the specific inhibitor HA15 improved the cytotoxicity of DPE against both cell lines. 

We found that the inhibition of UPR sensors PERK and IRE1alpha influenced CHOP expression and the antiproliferative effect of DPE in wt p53 RKO cells in an opposite way, while slightly affecting mutp53 SW480 cells. Interestingly, ATF6 inhibition by ceapin was the only treatment able to potentiate the cytostatic effect of DPE against both wt and mutp53 cells, suggesting that targeting of this UPR sensor could be a more widely usable anticancer strategy, particularly in combination with treatments inducing ER stress. Ceapin reduced the expression of the molecular chaperone BiP in both cell lines, which is not surprising when considering that BiP is one of the ATF6 targets. The downregulation of BiP could be the mechanism through which ceapin further reduced colon cancer cell survival in combination with DPE, as indeed the use of HA15, BiP inhibitor, efficiently induced this effect. The induction of cell death by exacerbating ER stress in cancer cells, which are basally stressed compared to normal cells, or the manipulation of UPR sensor signaling to obtain this effect are emerging as promising anticancer strategies [29]. From previous studies, including those performed in our laboratory, it has emerged that UPR strongly influences processes that are deeply involved in cancerogenesis, such as cytokine release [30,31], EMT, angiogenesis [32,33], and anticancer immune response [7,34]. Oncogenes have been shown to activate UPR, such as in the case of c-myc, which through this mechanism promotes pro-survival autophagy [35], while mutp53 has been reported to sustain ATF6 activation to promote cancer cell survival [26]. Interestingly, in the present study, we found that ATF6, as activated by DPE, sustained mutp53 in SW480 cells, as indeed this molecule was strongly downregulated following ceapin treatment. Conversely, the combination of DPE and ceapin resulted in the stabilization of wtp53 in RKO cells. Both wtp53 activation and mutp53 downregulation may lead to an impairment of cell survival, as wtp53 may induce cell cycle arrest or trigger apoptosis [36,37], while the reduction of mutp53 expression reduces cancer cell survival due to the fact that cancer cells become addicted to the effects mediated by this molecule, which acts as an oncogene [28]. Mutp53 is indeed able to cross-talk with several oncogenic pathways, which once activated by mutp53, strongly sustain its expression [38,39]. The data obtained in this study suggest that ATF6 and mutp53 may establish another interplay that is crucial for the survival and proliferation of stressed colon cancer cells. Another novel finding is that ATF6 restrains the activation of wtp53 in stressed cancer cells, which may be added to the pro-survival mechanisms induced by this UPR sensor. 

UPR has been found to be activated in most tumor samples originating from colon cancer biopsies, while the expression of UPR molecules such as BiP and PERK/eIF2 alpha and the XBP1 axis have been correlated with a poor prognosis. Moreover, treatments that exacerbate the basal ER stress or block the branches of the UPR are currently being used in in vitro and in vivo models to assess their impacts on growth and progression of CRC [40,41,42].

In conclusion, this study unveils new insights into the role of UPR sensor activation in the cytotoxic effects induced in colon cancer cells untreated or treated by DPE, a compound that has been demonstrated to be cytotoxic against colon cancer cells while being safe for normal epithelial colon cells. 

## Data Availability

The datasets generated and analyzed during the current study are available from the corresponding author upon reasonable request.

## References

[1] Wang M., Wey S., Zhang Y., Ye R., Lee A.S. (2009). Role of the unfolded protein response regulator GRP78/BiP in development, cancer, and neurological disorders. Antioxid. Redox Signal..

[2] Dong D., Ni M., Li J., Xiong S., Ye W., Virrey J.J., Mao C., Ye R., Wang M., Pen L. (2008). Critical role of the stress chaperone GRP78/BiP in tumor proliferation, survival, and tumor angiogenesis in transgene-induced mammary tumor development. Cancer Res..

[3] Cerezo M., Benhida R., Rocchi S. (2016). Targeting BIP to induce Endoplasmic Reticulum stress and cancer cell death. Oncoscience.

[4] Virrey J.J., Dong D., Stiles C., Patterson J.B., Pen L., Ni M., Schonthal A.H., Chen T.C., Hofman F.M., Lee A.S. (2008). Stress chaperone GRP78/BiP confers chemoresistance to tumor-associated endothelial cells. Mol. Cancer Res..

[5] Bianchi M.E. (2014). Killing cancer cells, twice with one shot. Cell Death Differ..

[6] Zitvogel L., Ayyoub M., Routy B., Kroemer G. (2016). Microbiome and Anticancer Immunosurveillance. Cell.

[7] Gilardini Montani M.S., Benedetti R., Piconese S., Pulcinelli F.M., Timperio A.M., Romeo M.A., Masuelli L., Mattei M., Bei R., D’Orazi G. (2021). PGE2 Released by Pancreatic Cancer Cells Undergoing ER Stress Transfers the Stress to DCs Impairing Their Immune Function. Mol. Cancer Ther..

[8] Martucciello S., Masullo M., Cerulli A., Piacente S. (2020). Natural Products Targeting ER Stress, and the Functional Link to Mitochondria. Int. J. Mol. Sci..

[9] Cirone M., Di Renzo L., Lotti L.V., Conte V., Trivedi P., Santarelli R., Gonnella R., Frati L., Faggioni A. (2012). Activation of dendritic cells by tumor cell death. Oncoimmunology.

[10] Guichard C., Pedruzzi E., Fay M., Marie J.C., Braut-Boucher F., Daniel F., Grodet A., Gougerot-Pocidalo M.A., Chastre E., Kotelevets L. (2006). Dihydroxyphenylethanol induces apoptosis by activating serine/threonine protein phosphatase PP2A and promotes the endoplasmic reticulum stress response in human colon carcinoma cells. Carcinogenesis.

[11] Martinez L., Ros G., Nieto G. (2018). Hydroxytyrosol: Health Benefits and Use as Functional Ingredient in Meat. Medicines.

[12] Strate L.L., Syngal S. (2005). Hereditary colorectal cancer syndromes. Cancer Causes Control.

[13] Dekker E., Tanis P.J., Vleugels J.L.A., Kasi P.M., Wallace M.B. (2019). Colorectal cancer. Lancet.

[14] Wang Y., Shen J., Arenzana N., Tirasophon W., Kaufman R.J., Prywes R. (2000). Activation of ATF6 and an ATF6 DNA binding site by the endoplasmic reticulum stress response. J. Biol. Chem..

[15] Hetz C., Zhang K., Kaufman R.J. (2020). Mechanisms, regulation and functions of the unfolded protein response. Nat. Rev. Mol. Cell Biol..

[16] Jo H.J., Yang J.W., Park J.H., Choi E.S., Lim C.S., Lee S., Han C.Y. (2019). Endoplasmic Reticulum Stress Increases DUSP5 Expression via PERK-CHOP Pathway, Leading to Hepatocyte Death. Int. J. Mol. Sci..

[17] Buffet C., Hecale-Perlemoine K., Bricaire L., Dumont F., Baudry C., Tissier F., Bertherat J., Cochand-Priollet B., Raffin-Sanson M.L., Cormier F. (2017). DUSP5 and DUSP6, two ERK specific phosphatases, are markers of a higher MAPK signaling activation in BRAF mutated thyroid cancers. PLoS ONE.

[18] Darling N.J., Cook S.J. (2014). The role of MAPK signalling pathways in the response to endoplasmic reticulum stress. Biochim. Biophys. Acta.

[19] Gomez-Bougie P., Halliez M., Moreau P., Pellat-Deceunynck C., Amiot M. (2016). Repression of Mcl-1 and disruption of the Mcl-1/Bak interaction in myeloma cells couple ER stress to mitochondrial apoptosis. Cancer Lett..

[20] Cullinan S.B., Zhang D., Hannink M., Arvisais E., Kaufman R.J., Diehl J.A. (2003). Nrf2 is a direct PERK substrate and effector of PERK-dependent cell survival. Mol. Cell Biol..

[21] Meares G.P., Liu Y., Rajbhandari R., Qin H., Nozell S.E., Mobley J.A., Corbett J.A., Benveniste E.N. (2014). PERK-dependent activation of JAK1 and STAT3 contributes to endoplasmic reticulum stress-induced inflammation. Mol. Cell Biol..

[22] Rozpedek W., Pytel D., Mucha B., Leszczynska H., Diehl J.A., Majsterek I. (2016). The Role of the PERK/eIF2alpha/ATF4/CHOP Signaling Pathway in Tumor Progression During Endoplasmic Reticulum Stress. Curr. Mol. Med..

[23] Shuda M., Kondoh N., Imazeki N., Tanaka K., Okada T., Mori K., Hada A., Arai M., Wakatsuki T., Matsubara O. (2003). Activation of the ATF6, XBP1 and grp78 genes in human hepatocellular carcinoma: A possible involvement of the ER stress pathway in hepatocarcinogenesis. J. Hepatol..

[24] Yang H., Niemeijer M., van de Water B., Beltman J.B. (2020). ATF6 Is a Critical Determinant of CHOP Dynamics during the Unfolded Protein Response. iScience.

[25] Chen Y., Brandizzi F. (2013). IRE1: ER stress sensor and cell fate executor. Trends Cell Biol..

[26] Sicari D., Fantuz M., Bellazzo A., Valentino E., Apollonio M., Pontisso I., Di Cristino F., Dal Ferro M., Bicciato S., Del Sal G. (2019). Mutant p53 improves cancer cells’ resistance to endoplasmic reticulum stress by sustaining activation of the UPR regulator ATF6. Oncogene.

[27] Luo Q., Beaver J.M., Liu Y., Zhang Z. (2017). Dynamics of p53: A Master Decider of Cell Fate. Genes.

[28] Mantovani F., Collavin L., Del Sal G. (2019). Mutant p53 as a guardian of the cancer cell. Cell Death Differ..

[29] Ojha R., Amaravadi R.K. (2017). Targeting the unfolded protein response in cancer. Pharmacol. Res..

[30] Smith J.A. (2018). Regulation of Cytokine Production by the Unfolded Protein Response; Implications for Infection and Autoimmunity. Front. Immunol..

[31] Gonnella R., Gilardini Montani M.S., Guttieri L., Romeo M.A., Santarelli R., Cirone M. (2021). IRE1 Alpha/XBP1 Axis Sustains Primary Effusion Lymphoma Cell Survival by Promoting Cytokine Release and STAT3 Activation. Biomedicines.

[32] Santamaria P.G., Mazon M.J., Eraso P., Portillo F. (2019). UPR: An Upstream Signal to EMT Induction in Cancer. J. Clin. Med..

[33] Santarelli R., Arteni A.M.B., Gilardini Montani M.S., Romeo M.A., Gaeta A., Gonnella R., Faggioni A., Cirone M. (2020). KSHV dysregulates bulk macroautophagy, mitophagy and UPR to promote endothelial to mesenchymal transition and CCL2 release, key events in viral-driven sarcomagenesis. Int. J. Cancer.

[34] Cubillos-Ruiz J.R., Bettigole S.E., Glimcher L.H. (2017). Tumorigenic and Immunosuppressive Effects of Endoplasmic Reticulum Stress in Cancer. Cell.

[35] Hart L.S., Cunningham J.T., Datta T., Dey S., Tameire F., Lehman S.L., Qiu B., Zhang H., Cerniglia G., Bi M. (2012). ER stress-mediated autophagy promotes Myc-dependent transformation and tumor growth. J. Clin. Investig..

[36] Aubrey B.J., Kelly G.L., Janic A., Herold M.J., Strasser A. (2018). How does p53 induce apoptosis and how does this relate to p53-mediated tumour suppression?. Cell Death Differ..

[37] Fridman J.S., Lowe S.W. (2003). Control of apoptosis by p53. Oncogene.

[38] D’Orazi G., Cirone M. (2019). Mutant p53 and Cellular Stress Pathways: A Criminal Alliance That Promotes Cancer Progression. Cancers.

[39] D’Orazi G., Cordani M., Cirone M. (2021). Oncogenic pathways activated by pro-inflammatory cytokines promote mutant p53 stability: Clue for novel anticancer therapies. Cell Mol. Life Sci..

[40] Huang J., Pan H., Wang J., Wang T., Huo X., Ma Y., Lu Z., Sun B., Jiang H. (2021). Unfolded protein response in colorectal cancer. Cell Biosci..

[41] Walczak A., Gradzik K., Kabzinski J., Przybylowska-Sygut K., Majsterek I. (2019). The Role of the ER-Induced UPR Pathway and the Efficacy of Its Inhibitors and Inducers in the Inhibition of Tumor Progression. Oxid. Med. Cell. Longev..

[42] Fujimoto T., Yoshimatsu K., Watanabe K., Yokomizo H., Otani T., Matsumoto A., Osawa G., Onda M., Ogawa K. (2007). Overexpression of human X-box binding protein 1 (XBP-1) in colorectal adenomas and adenocarcinomas. Anticancer Res..

